# Early Mortality Following Systemic Anticancer Therapy in Lung Cancer: A Bayesian Spatiotemporal Multilevel Analysis

**DOI:** 10.1002/iid3.70368

**Published:** 2026-02-17

**Authors:** Getayeneh Antehunegn Tesema, Rob G. Stirling, Win Wah, Zemenu Tadesse Tessema, Stephane Heritier, Arul Earnest

**Affiliations:** ^1^ School of Public Health and Preventive Medicine Monash University Melbourne Victoria Australia; ^2^ Department of Epidemiology and Biostatistics, Institute of Public Health, College of Medicine and Health Sciences University of Gondar Gondar Ethiopia; ^3^ Central Clinical School, Faculty of Medicine, Nursing and Health Sciences Monash University Melbourne Victoria Australia; ^4^ Department of Respiratory Medicine Alfred Health Melbourne Victoria Australia; ^5^ Monash Centre for Occupational and Environmental Health, School of Public Health and Preventive Medicine Monash University Melbourne Victoria Australia

**Keywords:** Bayesian multilevel modeling, early mortality, lung cancer, spatiotemporal analysis, systemic anticancer therapy, Victoria

## Abstract

**Introduction:**

Despite advances in lung cancer treatment, early mortality following Systemic Anticancer Therapy (SACT) remains a major concern. Identifying patients at high risk of early mortality may inform treatment decision‐making, particularly where SACT may offer limited benefit. We aimed to quantify spatiotemporal patterns of early mortality following SACT and to examine the relative contributions of individual‐ and area‐level risk factors.

**Methods:**

We conducted a secondary analysis of data from the Victorian Lung Cancer Registry. Bayesian spatiotemporal multilevel models were used to assess associations between individual‐ and area‐level factors and early mortality following SACT. Adjusted Odds Ratios (AORs) with 95% Credible Interval (Crl) were reported to indicate statistical significance.

**Results:**

Substantial spatiotemporal variation in early mortality following SACT was observed across Victoria. Factors associated with increased odds of early mortality included age ≥ 60 years (AOR = 1.06, 95% Crl: 1.01–1.22), clinical stage II (AOR = 1.15, 95% Crl: 1.01–1.59), stage III (AOR = 1.28, 95% Crl: 1.01–2.05), stage IV (AOR = 3.19, 95% Crl: 2.12–5.07), comorbidity (AOR = 1.12, 95% Crl: 1.01–1.34), and poor performance status (AOR = 3.09, 95% Crl: 2.24–4.24). Presentation at a multidisciplinary meeting (AOR = 0.66, 95% Crl: 0.52–0.88) and receipt of supportive care screening (AOR = 0.53, 95% Crl: 0.41–0.69) were associated with reduced odds of early mortality.

**Conclusion:**

Marked spatiotemporal variation in early mortality following SACT highlights the need for targeted, risk‐informed treatment strategies, particularly for patients with advanced disease, comorbidities, poor performance status, and those residing in high‐risk areas.

## Summary

Geographic and temporal variation in early mortality following Systemic Anticancer Therapy (SACT) identifies regions with disproportionately high mortality. Characterizing these patterns enables targeted interventions and more effective allocation of healthcare resources to populations at greatest risk. Spatiotemporal analysis also provides insights into underlying drivers of early mortality, treatment effectiveness, and opportunities to improve patient care and supportive services.

Methodologically, this study enhances statistical power and reduces ecological bias by jointly modeling individual‐level covariates within a Bayesian spatiotemporal multilevel framework. By incorporating spatial and temporal random effects using an extended Hierarchical Related Regression approach, we demonstrate substantial spatiotemporal variation in early mortality following SACT across Victoria. The resulting risk maps highlight priority areas for further investigation and targeted interventions.

## Graphical abstract

This study enhances statistical power and addresses ecological biases by incorporating joint cross‐classifications of individual‐level covariates within a Bayesian spatiotemporal multilevel model. After accounting for both spatial and temporal random effects using the extended Hierarchical Related Regression framework, significant geographic variation in early mortality following Systemic Anticancer Therapy was observed in Victoria. These reported maps indicate potential areas for further research and targeted interventions.

## 1 Introduction

Lung cancer remains a major global health burden [1]. In 2020, more than 2.2 million new cases and nearly 1.8 million deaths were attributed to lung cancer worldwide, accounting for 18.4% of all cancer‐related deaths [2]. In Australia, lung cancer is among the most commonly diagnosed cancers and remains the leading cause of cancer‐related mortality [3]. Systemic Anticancer Therapy (SACT) is the recommended treatment modality for patients with advanced‐stage lung cancer [4]. Its delivery requires a comprehensive, patient‐centered approach that prioritizes therapeutic benefit while ensuring personalized treatment planning, close monitoring, and effective management of treatment‐related toxicities [5].

Despite its clinical benefits, SACT is associated with adverse outcomes, including an increased risk of death within 30 days of treatment initiation (early mortality) [6]. Evidence indicates that patients who die within 30 days of starting SACT are unlikely to derive meaningful benefit from treatment [7]. Consequently, 30‐day mortality is widely recognized as an important quality metric, reflecting both the safety and effectiveness of systemic therapies and potentially signaling deficiencies in patient selection, treatment protocols, or care delivery [8].

The Victorian Lung Cancer Registry (VLCR) uses early mortality, defined as death within 30 days following SACT, as a quality indicator to evaluate treatment outcomes and quality of care [9]. Studies from the Netherlands [7] and Australia [10] have reported early mortality rates of 6% and 5.6%, respectively, following the initiation of SACT. Several patient‐level predictors of early mortality have been identified, including age, sex, smoking status, clinical stage at diagnosis, treatment type, and tumor histology [7, 11, 12]. For example, Wallington et al. [11] reported that never smoking was associated with a reduced risk of early mortality, whereas male sex, receipt of palliative care, and advanced stage at diagnosis were associated with increased risk.

Beyond patient‐level risk factors, early mortality following SACT is also determined by area‑level factors, such as socioeconomic status and geographic remoteness. Patients from the most disadvantaged or remote areas experience a disproportionately higher risk of early mortality [11]. Moreover, lung cancer patients residing within the same geographic area and treatment period often share unmeasured contextual characteristics that induce spatial and temporal dependence. Failure to account for this dependence can lead to biased regression coefficients, underestimated uncertainty, and misspecified variance structures [13]. Traditional hierarchical or multilevel models generally assume independence between geographical areas [14], yet this assumption is frequently violated when adjacent regions exhibit spatial autocorrelation. In such cases, ignoring the underlying spatial dependency may produce inefficient estimators and incorrect inference. Incorporating spatial and temporal correlation structures is therefore essential to accurately estimate the underlying risk distribution.

Despite these methodological considerations, limited evidence exists on how much of the spatiotemporal variation in early mortality following SACT can be attributed simultaneously to individual‑ and area‑level determinants. To address this gap, we investigated the multilevel risk factors of early mortality following SACT using a Bayesian spatiotemporal model within the Hierarchical Related Regression (HRR) framework, allowing for flexible modeling of spatially structured effects, unstructured heterogeneity, and temporal trends.

## 2 Methods and Materials

### 2.1 Data Source and Study Design

This retrospective study utilized data from the VLCR, established in 2011 to monitor lung cancer care quality and patient‐reported outcomes. The study included patients diagnosed with lung cancer who received SACT between July 8, 2011, and May 24, 2022. Eligible patients were those who were treated with any combination of immunotherapy, targeted therapy (e.g., monoclonal antibodies and tyrosine kinase inhibitors), and chemotherapy, irrespective of the mode of administration. A total of 6704 lung cancer patients who underwent SACT were included in the study.

Individual‐level data were sourced from the VLCR, while area‐level data, including socioeconomic status and geographic remoteness, were obtained from the 2016 Australian Bureau of Statistics (ABS) [15]. Specifically, the Socioeconomic Index for Areas (SEIFA) and Accessibility/Remoteness Index for Australia plus (ARIA+) were used to assess socioeconomic status and geographic accessibility, respectively. The Local Government Area (LGA) was selected as the spatial unit of analysis, as it offers policy relevance and consistent access to relevant area‐level data. LGA shapefiles were retrieved from the 2021 ABS [16].

In Australia, LGAs represent the third‐level government division below states and territories. As of 2021, the median population size of an LGA was 12,905, with an Interquartile Range (IQR) of 42,872 [17]. Victoria is divided into 79 LGAs, 48 of which are classified as regional areas.

### 2.2 Outcome Measure and Covariates

The primary outcome was early mortality following SACT, defined as death within 30 days of initiating treatment, in accordance with Australian guidelines for lung cancer management [18].

Individual‐level covariates included demographic and clinical factors, such as age, smoking status, clinical stage at diagnosis, treatment intent, type of diagnosing hospital, received supportive care screening, Aboriginal and Torres Strait Islanders status, Eastern Cooperative Oncology Group (ECOG) performance status, and presented at a Multidisciplinary Meeting (MDM). The ECOG performance status, a standard measure of a patient's functional status, ranges from ECOG 0 (fully active) to ECOG 5 (dead) [19]. It was categorized as good (ECOG score < 2) or poor (ECOG score ≥ 2), based on the impact of the disease on daily living abilities.

Area‐level covariates included the Index of Socioeconomic Advantage and Disadvantage (IRSAD) and remoteness. IRSAD scores were grouped into quartiles, with the first quartile representing the most disadvantaged and the fourth quartile representing the least disadvantaged socioeconomic groups. Patients' residential LGA IRSAD scores were used as a proxy for socioeconomic status. Geographic remoteness was assessed using the ARIA+ index, which classifies areas into five levels of accessibility: very remote (ARIA+ score > 9.08), remote (ARIA+ score 5.80–9.08), moderately accessible (ARIA+ score 3.51–5.80), accessible (ARIA+ score 1.84–3.51), and highly accessible (ARIA+ score < 1.84) [20]. ARIA+ scores were utilized to assess the level of geographic remoteness and its potential impact on access to healthcare services.

### 2.3 Statistical Analysis

#### 2.3.1 Bayesian Spatiotemporal Multilevel Model

Data cleaning and management, statistical modeling, and mapping were conducted using STATA version 14, MultiBUGS version 2.0, and R version 4.3.3 for data cleaning and management, modeling, and mapping, respectively. The 12‐year study period (2011–2022) was divided into three intervals: 2011–2014, 2015–2018, and 2019–2022. This temporal aggregation ensured adequate sample sizes within each period and improved the statistical stability of parameter estimates.

The state of Victoria comprises 79 LGAs (indexed as j=1,2,…,79), with data available across three time periods (t=1,2,3). Although spatiotemporal models are widely used in cancer epidemiology, they are susceptible to confounding and identifiability issues, particularly when individual‐ and area‐level effects are not adequately disentangled.

To address these challenges, we applied an extension of the HRR framework developed by Jackson et al. [21] and Jonker et al. [22]. This framework enables the simultaneous analysis of individual‐ and aggregate‐level outcomes, together with individual‐ and area‐level risk factors, within a single modeling structure. By jointly modeling data across levels, the HRR approach reduces ecological bias arising from model misspecification and improves parameter identifiability compared with a conventional ecological model.

Within the HRR framework, we fitted a Bayesian spatiotemporal multilevel model to examine associations between individual‐ and area‐level risk factors and early mortality following SACT. This approach allows one to capture both the characteristics of individual patients and the features of the areas in which they live, providing a more accurate and policy‑relevant understanding of factors influencing early mortality. Compared with separate analyses of individual‐ and aggregate‐level data, this approach increases statistical power and reduces ecological bias attributable to confounding, limited information, or model misspecification.

A Conditional Autoregressive (CAR) prior was specified for the spatially structured random effects to capture spatial dependence, assuming that the random effect for a given LGA was correlated with those of neighboring LGAs. An Identically and Independently Distributed prior was assigned to the unstructured spatial random effects to account for residual heterogeneity. The spatially structured variance component (CAR prior) smoothed risk estimates towards the local mean of adjacent LGAs, while the unstructured variance component (assumed to follow a normal distribution) smoothed estimates towards the global mean across the study region.

Temporal variation was modeled using both linear and quadratic time terms to flexibly capture temporal trends in early mortality [23]. By incorporating within‐LGA variability in exposure and jointly modeling individual‐ and aggregated‐level outcomes, the HRR‐based spatiotemporal multilevel CAR model further enhanced statistical efficiency and mitigated ecological bias [24, 25].

#### 2.3.2 Individual‐Level Outcome Model

The individual‐level outcome $y_{ijt}$ (early mortality for an individual *i* in LGA *j* at time *t*) was modeled using a standard multilevel logistic regression model, accounting for the dependencies among individuals within the same area and time [26].

The model was specified as follows:

$$y_{ijt}\sim \mathrm{Bernoulli}\left(p_{ijt}\right),$$

(1) $$\text{logit}\left(p_{ijt}\right)=\mu +{\beta x}_{ijt}+{\gamma z}_{j}+u_{j}+t_{t},$$

where $t_{t}$ represents the temporal random effect, individual‐level predictors $x_{ij}$, area‐level predictors $z_{j}$, and $u_{j}$ were the spatial random effect, assumed to be normally distributed across LGAs with unknown mean μ and $\sigma^{2}$.

#### 2.3.3 Aggregate‐Level Outcome Model

The traditional ecological analysis directly models aggregated outcomes based on area‐level exposures via a logistic regression or count models [27]. However, ecological analysis is susceptible to ecological bias due to the within‐area variability in both exposures of interest and confounders [28]. Our model overcomes this limitation by including the joint within‐area distribution of covariates, modeled as

$$y_{jt}\sim \mathrm{Binomial}\left(N_{jt},P_{jt}\right),$$

(2) $$p_{jt}=\sum_{k}f_{jtk}p_{jtk}=\sum_{r_{1},r_{2},r_{3},r_{4},r_{5},r_{6}}f^{jt,r_{1},r_{2},r_{3},r_{4},r_{5},r_{6}}p^{jt,r_{1},r_{2},r_{3},r_{4},r_{5},r_{6}},$$

where $N_{jt}$ is the population at risk in LGA *j* during year *t*, $P_{jt}$ represents the average risk of early mortality for an individual in LGA *j* during year *t*, and $f_{jtk}$ is the joint within‐area distribution of individual covariates in LGA *j* during year *t*. The average risk of early mortality for an individual in LGA *j* during year *t* ($p_{jt}$) is determined by integrating the individual‐level model over the joint within‐area distribution of covariates [21, 29, 30].

As the data were aggregated at the LGA level, overdispersion was assessed to ensure the appropriate model was selected. We found that the overdispersion parameter was nonsignificant, indicating no substantial difference between the mean and variance. This finding led us to choose the Poisson model for the analysis. Additionally, we clarified the process for calculating the Deviance Information Criterion (DIC). The Poisson likelihood function was used for model fitting, and the DIC was calculated based on this likelihood function. The model with the lowest DIC value was selected, indicating the best‐fitting model for our data.

The Bayesian spatiotemporal multilevel model integrates the conditional probability of patients (equation a) over the joint within‐area distribution of individual covariates ($f_{jtk}$). To reduce the ecological bias and estimate the independent effects of individual‐ and area‐level risk factors, we calculated the within‐area variability of covariates ($f_{jtk}$) from multiple individual‐level covariates. Here, $f_{jtk}$ represents the probability that an individual in area *j* occupies year *t* and category *k*. The category *k* specifies the possible combination of individual‐level covariates, including age (*r*_1_), MDM meeting (*r*_2_), supportive care screening (*r*_3_), clinical stage at diagnosis (*r*_4_), comorbidity (*r*_5_), and ECOG performance status (*r*_6_). The combinations yield *k* = *r*_1_, *r*_2_, *r*_3_, *r*_4_, *r*_5_, *r*_6_ = 2 × 2 × 2 × 4 × 2 × 3, where *r*_1_ has two categories, …, *r*_6_ has three categories.

The HRR model simultaneously incorporates both the individual‐level data, including covariates and outcomes, using the standard logistic model, and the aggregate outcome data from the corresponding LGAs through a marginal model. The full likelihood is the product of the likelihoods of individual‐ and aggregate‐level outcome models.

The Bayesian spatiotemporal multilevel model was expressed as

$$\text{logit}(p_{j,t,r_{1},r_{2},r_{3},r_{4},r_{5},r_{6})}=\mu +BX_{jtk}+{\gamma Z}_{j}+u_{j}+v_{j}+t_{t}+t_{t}^{2}.$$

Structured spatial random effects ($u_{j}$) and unstructured random effects ($v_{j}$) were modeled using CAR and IDD, respectively. The model considered the linear ($t_{t}$) and quadratic ($t_{t}^{2}$) temporal effects specified to capture both linear and nonlinear temporal variations in the log‐odds of early mortality.

#### 2.3.4 Model Implementation

A Bayesian approach was employed for all parameter estimations. Assuming a 95% probability that the true estimate (Odds Ratio) lies between 0.1 and 10, we assigned weakly informative priors to regression coefficients (*β*) and intercept (μ) parameters. Specifically, we used Normal (0, 0.725) priors for *β* and an improper flat prior for μ. A CAR prior distribution was assigned for the spatially structured random effect [31]. The choices of priors, initial values, and hyperparameters were guided by sensitivity analyses. Various combinations of priors with different distributions and parameter values were compared and assessed in terms of model convergence, utilizing visual diagnostics and Brooks Gelman Rubin (BGR) statistics [32]. Additional convergence checks, including the Monte Carlo (MC) error, were computed to the accuracy of estimates [33].

For the spatial dependence term, we utilized the CAR model developed by Besag:

$$\left[u_{i}|u_{j},i\neq j,{\tau_{u}}^{2}\right]\sim N\left({ū}_{i,}{\tau_{i}}^{2}\right),$$

$$\text{where }{ū}_{i}=\pi \frac{1}{\sum_{j}w_{ij}}\sum u_{j}w_{ij},$$

$${\tau_{i}}^{2}=\frac{{\tau_{u}}^{2}}{\sum_{j}w_{ij}}.$$

Here, *w_ij_* = 1 if *i* and *j* are adjacent, and 0 otherwise. To define the neighborhood adjacency matrix, we applied the Queen‐1 method, including all immediate neighbors that share a common border. Each neighbor contributed equally to the adjacency weight matrix.

All the models were analyzed using MultiBUGS (Version 2.0, University of Cambridge, UK) using the Markov Chain Monte Carlo (MCMC) sampling technique. The MCMC algorithm was executed for 100,000 iterations, discarding 50,000 as burn‐in. We fitted both bivariable and multivariable Bayesian spatiotemporal multilevel CAR models. Variables were considered for inclusion in the multivariable model based on their statistical significance in bivariable analyses and their clinical relevance, as supported by existing literature. Model development and selection were guided by the DIC [34], with preference given to models demonstrating lower DIC values. Model building commenced with the variable associated with the lowest DIC, followed by the sequential addition of variables ranked by importance. A variable was retained in the model only if its inclusion resulted in a reduction of at least five points in the DIC, indicating meaningful improvement in model fit.

In the final multivariable model, results were reported as Adjusted Odds Ratio (AOR) with corresponding 95% Credible Interval (Crl) to quantify the strength of associations and statistical uncertainty.

## 3 Results

### 3.1 Characteristics of the Study Participants

A total of 6704 lung cancer patients receiving SACT were included in this study. Of these, 5429 (80.98%) were diagnosed with Nonsmall Cell Lung Cancer (NSCLC). Approximately 76.88% of patients were aged over 60 years, 35.78% were current smokers, and 56.80% were male. Additionally, 4174 patients (62.26%) had a good ECOG performance status. More than half of the patients (56.18%) were diagnosed at stage IV. Regarding area‐level remoteness and IRSAD, the largest proportion of patients resided in highly accessible LGAs and in the least disadvantaged quartile (Table 1).

**Table 1:** Demographic and clinical characteristics of lung cancer patients receiving systemic anticancer treatment in Victoria (2011–2022).

Variables	Categories	Frequency (*n* = 6704)	Percentage (%)
Cancer type	NSCLC	5429	80.98
	SCLC	1275	19.02
Sex	Male	3808	56.80
	Female	2896	43.20
Age (in years)	≤ 60	1550	23.12
	> 60	5154	76.88
Smoking status	Current smoker	2399	35.78
	Ex‐smoker	3313	49.42
	Never	836	12.47
	Not stated	156	2.33
ATSI status	ATSI	66	0.98
	Non‐ATSI	6565	97.93
	Unknown	73	1.03
Reviewed at the MDM	No	2257	33.67
	Yes	4447	66.33
Screened for supportive care	No	3726	55.58
	Yes	2978	44.42
ECOG performance status	Good	4174	62.26
	Poor	660	9.84
	Not stated	1870	27.89
Having comorbidity	No	3184	47.49
	Yes	3520	52.51
Surgery performed	No	5560	82.90
	Yes	1144	17.06
Clinical stage at diagnosis	Stage I	199	2.97
	Stage II	471	7.03
	Stage III	1401	20.90
	Stage IV	3766	56.18
	Not stated	867	12.93
Treatment intent	Adjuvant	755	11.27
	Radical	1102	16.44
	Palliative	3567	53.22
	Not stated	1278	19.07
Area‐level socioeconomic status	Most disadvantage	1098	16.38
	Second quartile	964	14.38
	Third quartile	2161	32.23
	Least disadvantaged	2481	37.01
Geographic remoteness	Accessible	545	8.13
	Moderately accessible	194	2.89
	Highly accessible	5965	88.98

Abbreviations: ATSI, Aboriginal and Torres Strait Islander; ECOG, Eastern Cooperative Oncology Group; MDM, Multidisciplinary Meeting; NSCLC, Nonsmall Cell Lung Cancer; SCLC, Small Cell Lung Cancer.

### 3.2 Early Mortality Following SACT

The early mortality rate following SACT was 4.25% (95% CI: 3.79–4.76). The early mortality rate was 4.64% among patients aged over 60 years. Lower early mortality rates were observed among patients whose cases were presented at an MDM (3.55%) and among those who received supportive care screening (3.19%). In contrast, patients with poor ECOG performance status exhibited substantially higher early mortality (10.15%) (Table 2).

**Table 2:** Distribution of lung cancer patients receiving systemic anticancer treatment by patient characteristics.

	Early mortality following SACT		*p* value
Variables	No	Yes	
*Lung cancer types*			
NSCLC	5210 (95.97)	219 (4.03)	0.069
SCLC	1209 (94.82)	66 (5.18)	
*Age (in years)*			
≤ 60	1691 (96.85)	55 (3.15)	0.008
> 60	4728 (95.36)	230 (4.64)	
*Area‐level socioeconomic status*			
Most disadvantaged	2377 (94.97)	126 (5.03)	0.049
Second quartile	1006 (95.63)	46 (4.37)	
Third quartile	2075 (96.01)	86 (3.98)	
Least disadvantaged	1063 (96.81)	35 (3.19)	
*Presented at MDM*			
No	2130 (94.37)	127 (5.63)	< 0.0001
Yes	4289 (96.45)	158 (3.55)	
*Received supportive care screening*			
No	3536 (94.90)	190 (5.10)	< 0.0001
Yes	2883 (96.81)	95 (3.19)	
*ECOG performance status*			
Good	4063 (97.34)	111 (2.66)	< 0.0001
Poor	593 (89.85)	67 (10.15)	
Not stated	1763 (94.28)	107 (5.72)	
*Clinical stage at diagnosis*			
Stage I	198 (99.50)	1 (0.50)	< 0.0001
Stage II	469 (99.58)	2 (0.42)	
Stage III	1376 (98.22)	25 (1.78)	
Stage IV	3547 (94.18)	219 (5.82)	
Not stated	829 (95.62)	38 (4.38)	
*Had comorbidity*			
No	3071 (96.45)	113 (3.55)	0.007
Yes	3348 (95.11)	172 (4.89)	
Total	6419 (95.75)	285 (4.25)	

Abbreviations: ECOG, Eastern Cooperative Oncology Group; MDM, Multidisciplinary Meeting; NSCLC, Nonsmall Cell Lung Cancer; SACT, Systemic Anticancer Therapy; SCLC, Small Cell Lung Cancer.

### 3.3 Individual‐ and Area‐Level Risk Factors

To ensure the robustness of the estimates, model convergence was assessed using various diagnostic tests, including the BGR statistics plot, which confirmed that all parameters had converged (Figure S1). All parameters demonstrated MC errors below the 5% threshold. Variables that were statistically significant in the bivariable analysis were included in the multivariable model, with less important variables removed stepwise based on their significance and the DIC.

In the final best‐fitting model, age ≥ 60 years, advanced clinical stage, the presence of comorbidities, and poor ECOG performance status were significantly associated with an increased odds of early mortality following SACT. In contrast, presentation at MDM and receiving supportive care screening were significantly associated with a decreased risk of early mortality following SACT.

Patients aged over 60 years had a higher odds of early mortality (AOR = 1.06, 95% Crl: 1.00–1.22). The odds of early mortality were reduced by 34% among patients presented at MDM (AOR = 0.66, 95% Crl: 0.52–0.88). Additionally, patients who received supportive care screening had a lower odds of early mortality (AOR = 0.53, 95% Crl: 0.41–0.69). Compared with patients at clinical stage I, the odds of early mortality were 1.15 times higher for those at stage II (AOR = 1.15, 95% Crl: 1.01–1.59), 1.28 times higher at stage III (AOR = 1.28, 95% Crl: 1.01–2.05), and 3.19 times higher at stage IV (AOR = 3.19, 95% Crl: 2.12–5.07). Moreover, having an underlying comorbidity increased the odds of early mortality by 12% (AOR = 1.12, 95% Crl: 1.01–1.34), and patients with poor ECOG performance status had 3.09 times higher odds of early mortality (AOR = 3.09, 95% Crl: 2.24–4.24) (Table 3).

**Table 3:** Unadjusted and adjusted odds ratio estimates of early mortality following systemic anticancer therapy.

Variables	Unadjusted odds ratio with 95% Crl	AOR with 95% Crl
*Age (in years)*		
≤ 60	1	1
> 60	1.20 (1.01, 1.53)*	1.06 (1.01, 1.22)*
*Presented at an MDM*		
No	1	1
Yes	0.50 (0.49, 0.51)*	0.66 (0.52, 0.88)*
*Received supportive care screening*		
No	1	1
Yes	0.27 (0.22, 0.32)*	0.53 (0.41, 0.69)*
*Clinical stage at diagnosis*		
Stage I	1	1
Stage II	1.04 (1.01, 1.13)*	1.15 (1.01, 1.59)*
Stage III	1.02 (1.01, 1.06)*	1.28 (1.01, 2.05)*
Stage IV	1.15 (1.01, 1.38)*	3.19 (2.12, 5.07)*
*Had comorbidity*		
No	1	1
Yes	1.01 (1.00, 1.02)*	1.12 (1.01, 1.34)*
*ECOG performance status*		
Good	1	1
Poor	3.61 (2.61, 4.85)*	3.09 (2.24, 4.24)*

*Note:* An unadjusted odds ratio was obtained from the bivariable Bayesian spatiotemporal multilevel model, whereas an AOR was obtained from the multivariable spatiotemporal multilevel models.

Abbreviations: AOR, adjusted odds ratio; Crl, Credible Interval; DIC, Deviance Information Criteria; ECOG, Eastern Cooperative Oncology Group; MDM, Multidisciplinary Meeting; OR, Odds Ratio.

* Statistically significant (95% Crl did not contain the null value [OR = 1]).

### 3.4 Spatiotemporal Variation of Early Mortality Following SACT

**Figure 1:**
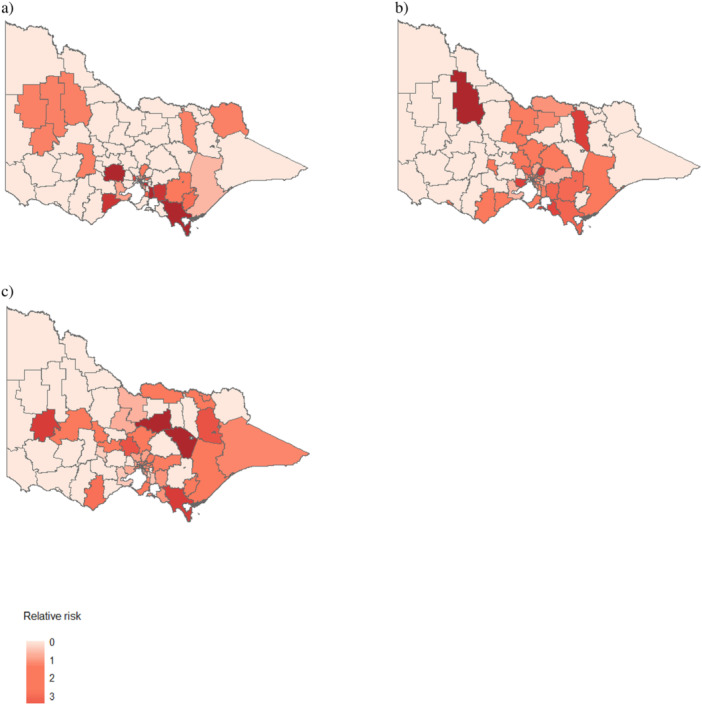
Standardized relative risk of early mortality following systemic anticancer therapy (SACT) among lung cancer patients in Victoria, Australia. This figure presents maps showing the relative risk of early mortality following SACT across three time periods: (a) 2011–2014, (b) 2015–2018, and (c) 2019–2022. The relative risk was estimated at the local government area level. Areas shaded with more intense colors indicate a higher risk of early mortality, while areas with lighter shading indicate a lower risk.

The standardized relative risk of early mortality following SACT among lung cancer patients showed substantial spatiotemporal variation across LGAs in Victoria (Figure 1). LGAs such as South Gippsland, Surf Coast, Greater Geelong, and Melbourne consistently exhibited a higher risk of early mortality, whereas those in the western and north‐western regions consistently showed a lower risk. Approximately 87.65% of the spatial variation in early mortality following SACT was attributed to structured spatial risk factors.

## 4 Discussion

The early mortality rate following SACT among patients with lung cancer was found to be 4.25%. We observed substantial geographic and temporal variations in early mortality among lung cancer patients in Victoria. Clinical stage at diagnosis, age, ECOG performance status, comorbidities, presentation at an MDM, and receipt of supportive care screening were all significantly associated with early mortality.

The incidence of early mortality in this study was lower than that reported in a study from London, UK [35], but higher than findings from a previous study conducted in Australia [36]. These discrepancies may be attributed to differences in the study populations and operational definitions used to define early mortality following SACT. For instance, the study by Yoong et al. [36] focused on a single regional hospital in Victoria, including only 378 cancer patients who received chemotherapy or targeted therapy, excluding immunotherapy. In contrast, our multicenter study included 6704 patients who received SACT from 2011 onwards, with SACT encompassing all chemotherapy, targeted biological therapy, and immunotherapy. The observed spatiotemporal variation in early mortality may reflect disparities in the distribution of healthcare facilities and access to specialized cancer care [37, 38]. Additionally, differences in socioeconomic status, insurance coverage, and environmental factors could further contribute to these variations in mortality rates.

The advanced clinical stage was significantly associated with an increased risk of early mortality. This finding is consistent with previous studies [12, 39], likely due to the more aggressive and widespread nature of the disease at diagnosis, which complicates treatment and reduces responsiveness to therapy [40]. Besides, patients with advanced‐stage lung cancer may not qualify for curative treatments, often receiving only palliative care, which may be inadequate to control the cancer progression [41, 42]. Such factors could justify the risk of early mortality following SACT in advanced‐stage lung cancer. As supported by published studies [43, 44], poor ECOG performance status was linked to an increased risk of early mortality. Poor ECOG performance status is associated with reduced functional capacity and significant physical limitations, which adversely affect overall health [45]. Typically, these patients have underlying medical conditions that increase their vulnerability to drug toxicities [46].

Patients aged over 60 years were found to be at a higher risk of early mortality, consistent with findings from other studies [10, 39]. This may be attributable to older patients' reduced ability to tolerate the toxic effects of SACT, as they often present with comorbid conditions and decreased organ function [47, 48]. Participation in MDT meetings significantly lowered the risk of early mortality, a finding supported by a previous study [8]. The comprehensive evaluation of each patient's case by the MDT team enables informed treatment decisions, thereby potentially reducing mortality risk [49].

Consistent with the prior study [50], screening for supportive care significantly reduced the risk of early mortality following SACT. Lung cancer patients frequently experience anxiety, depression, stress, and other emotional challenges [51]. Therefore, providing supportive care can enhance patient survival. This study also identified a significant impact of comorbidities on early mortality after SACT, corroborating findings from previous research [8, 11]. Managing patients with comorbidities is often more complex, necessitating additional medications that increase the risk of treatment‐related side effects, including toxicity, infections, and drug‐drug interactions [52].

This study employed a Bayesian spatiotemporal multilevel modeling approach, which helped mitigate ecological bias and enhanced statistical power [53]. The use of large, multicentre data from the VLCR further strengthened the robustness and generalizability of the findings within the covered population. Additionally, the modeling framework applied here can be adapted to other settings to inform targeted intervention strategies.

However, several limitations must be acknowledged. First, the potential for residual confounding remains, as certain clinically relevant variables, such as patient frailty, preferences, and treatment refusals, were not available in the VLCR. Furthermore, important structural and healthcare access‐related factors, including disparities in service availability and provider density, could not be incorporated due to data limitations. Second, the retrospective nature of the study precludes causal inference. Finally, while the VLCR provides approximately 90% coverage of the target population, the findings may not be fully generalizable to regions or patient groups not captured in the registry.

Despite these limitations, this study provides a critical foundation for future research. The observed spatiotemporal patterns of early mortality following SACT offer valuable insights and can guide geographically focused case‐control or mixed‐methods studies aimed at identifying underlying structural, environmental, and clinical determinants.

## 5 Conclusion

This study reveals a concerning early mortality rate of 4.25% following SACT for lung cancer, with substantially higher risks observed among older patients, those with comorbidities, advanced‐stage disease, and poor performance status. Notably, presentation at MDM and receiving supportive care screening significantly lowers the odds of early mortality, highlighting the life‐saving potential of these interventions. These findings underscore an urgent need for targeted, region‐specific strategies to ensure equitable access to timely, guideline‐concordant lung cancer care across all LGAs.

## Author Contributions

**Getayeneh Antehunegn Tesema:** conceptualization, data curation, formal analysis, investigation, methodology, software, validation, visualization, writing – original draft, writing – review and editing. **Rob G. Stirling:** conceptualization, data curation, investigation, software, supervision, validation, visualization, writing – review and editing. **Win Wah:** conceptualization, data curation, formal analysis, investigation, methodology, software, supervision, validation, visualization, writing – review and editing. **Zemenu Tadesse Tessema:** conceptualization, data curation, formal analysis, methodology, software, supervision, validation, visualization, writing – review and editing. **Stephane Heritier:** conceptualization, data curation, investigation, methodology, software, supervision, validation, visualization, writing – review and editing. **Arul Earnest:** conceptualization, data curation, formal analysis, investigation, methodology, software, supervision, validation, visualization, writing – review and editing.

## Ethics Statement

The data collection and analysis were conducted according to the principles of the Declaration of Helsinki. The retrospective study protocol was reviewed and approved by the Human Research Ethics Committee of Monash University (approval number 35288). Hence, written informed consent was waived because of the retrospective nature of the study.

## Conflicts of Interest

The authors declare no conflicts of interest.

## Supporting information


**Figure S1:** Brooks‐Gelman‐Rubin diagnostic plot to assess convergence of model parameters To ensure the robustness of the estimates, we assessed model convergence using a BGR plot, confirming that all the parameters converged.

## Data Availability

The data that support the findings of this study are available on request from the corresponding author. The data are not publicly available due to privacy or ethical restrictions.
