# Supplementary figures and images for: Early Mortality Following Systemic Anticancer Therapy in Lung Cancer: A Bayesian Spatiotemporal Multilevel Analysis
Source: Immun Inflamm Dis. 2026 Feb 17;14(2):e70368. doi: 10.1002/iid3.70368 (PMC12914077; doi:10.1002/iid3.70368)

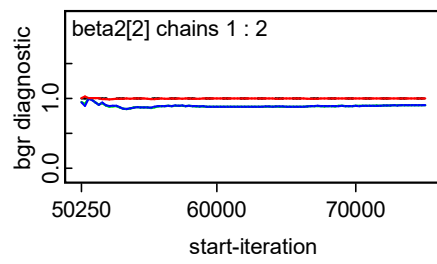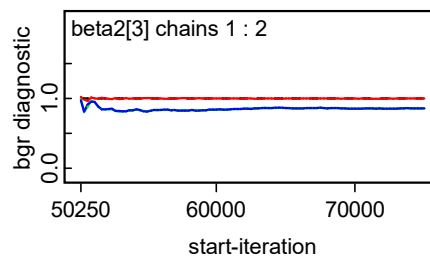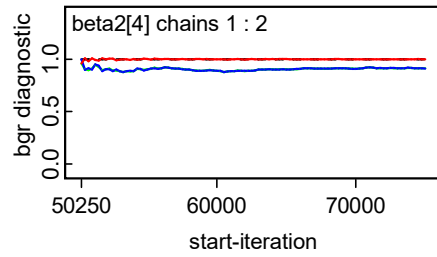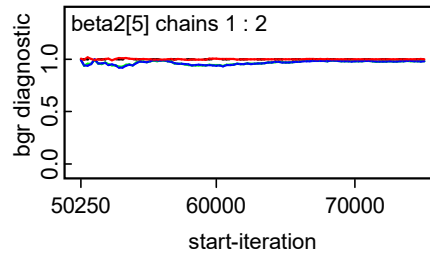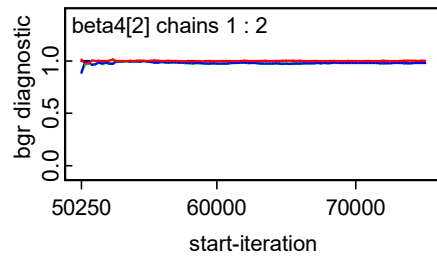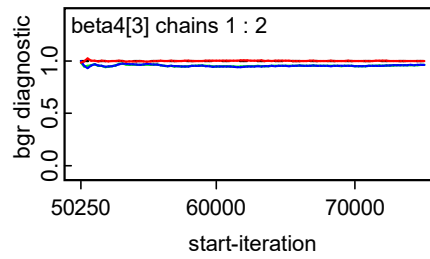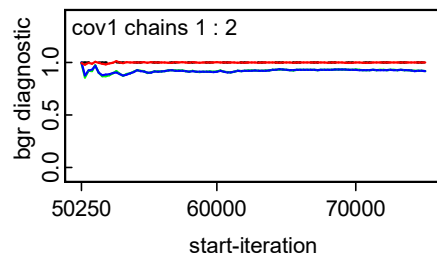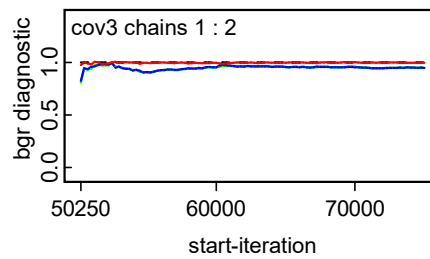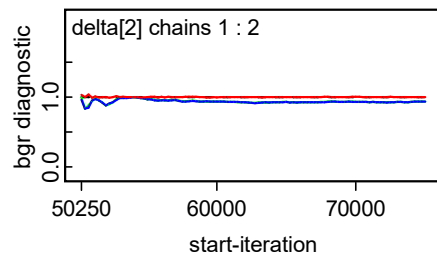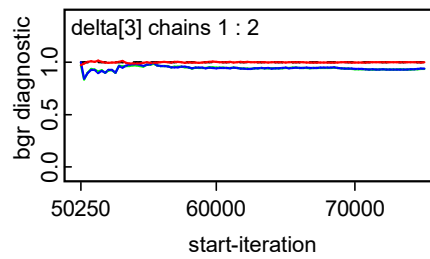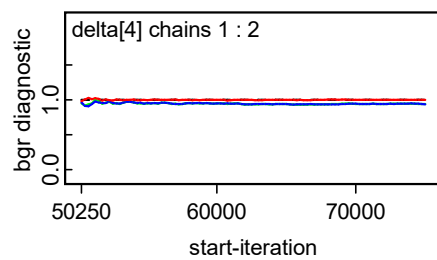

Supplement: Supplementary file 1 — Figure S1: Brooks‐Gelman‐Rubin diagnostic plot to assess convergence of model parameters To ensure the robustness of the estimates, we assessed model convergence using a BGR plot, confirming that all the parameters converged. [file IID3-14-e70368-s001.pdf]
